# A profile of The Clinical Course of Cognition and Comorbidity in Mild Cognitive Impairment and Dementia Study (The 4C study): two complementary longitudinal, clinical cohorts in the Netherlands

**DOI:** 10.1186/s12883-016-0750-9

**Published:** 2016-11-25

**Authors:** Weiqi Liao, Renske E. G. Hamel, Marcel G. M. Olde Rikkert, Saskia M. Oosterveld, Pauline Aalten, Frans R. J. Verhey, Philip Scheltens, Nicole Sistermans, Yolande A. L. Pijnenburg, Wiesje M. van der Flier, Inez H. G. B. Ramakers, René J. F. Melis

**Affiliations:** 1 Radboud Institute of Health Sciences, Department of Geriatric Medicine & Radboudumc Alzheimer Centre, Radboud University Medical Center, PO Box 9109, (House post 925), 6500 HB Nijmegen, The Netherlands; 2 Alzheimer Centre Limburg, School for Mental Health & Neuroscience, Maastricht University Medical Centre, Maastricht, The Netherlands; 3 Donders Institute for Brain, Cognition and Behaviour, Department of Geriatric Medicine & Radboudumc Alzheimer Centre, Radboud University Medical Center, Nijmegen, The Netherlands; 4 Alzheimer Center & Department of Neurology, VU University Medical Center, Neuroscience Campus, Amsterdam, The Netherlands; 5 Alzheimer Center & Department of Neurology, Department of Epidemiology and Biostatistics, VU University Medical Center, Amsterdam, The Netherlands

**Keywords:** Mild Cognitive Impairment (MCI), Dementia, Disease progression, Cognition, Comorbidity, Frailty

## Abstract

**Background:**

Heterogeneous disease trajectories of mild cognitive impairment (MCI) and dementia are frequently encountered in clinical practice, but there is still insufficient knowledge to understand the reasons and mechanisms causing this heterogeneity. In addition to correlates of the disorder, patient characteristics such as their health status, social environment, comorbidities and frailty may contribute to variability in trajectories over time. The current paper outlines the study design and the study population of and provides an overview of the data collected in the *Clinical Course of Cognition and Comorbidity in Mild Cognitive Impairment* (*4C-MCI cohort, n = 315) and Dementia* (*4C-Dementia cohort, n = 331) Study.*

**Methods:**

The two complementary longitudinal cohorts part of the 4C study began enrolment in March 2010. Participants were prospectively recruited from three collaborating Dutch Alzheimer Centers, with three annual follow-up assessments after baseline. Extensive neuropsychological assessments, and detailed profiling of comorbidities, health and frailty at each follow up were the key features of the 4C study. As such, the 4C study was designed to study if and how patients’ comorbidities and frailty are associated with the course of MCI and dementia measured with a comprehensive and multidimensional set of outcomes including cognition, daily functioning, quality of life, behavioral disturbances, caregiver burden, institutionalization and death and whether the effects of medical health and frailty differ between MCI and dementia stages of cognitive disorders.

**Conclusion:**

Sampled in a clinical setting, the 4C study complements population-based studies on neurodegenerative disorders in terms of the type of assessment (e.g. comorbidity, frailty, and functional status were repeatedly assessed). The 4C study complements available clinical cohorts of MCI and dementia patients, because the exclusion criteria were kept to a minimum, to obtain a sample that is representative for the average patient visiting a memory clinic.

## Background

Dementia is a syndrome of impaired cognitive functioning that interferes with living independently. It is a global public health issue with numbers of people suffering from dementia estimated to increase from 46.8 million in 2015 to 74.7 million in 2030, and to 131.5 million in 2050 worldwide [1]. Mild cognitive impairment (MCI) is a state of impaired cognitive performance that does not significantly interfere with independent living and is a risk factor for developing dementia. Both in MCI and dementia, progression over time varies tremendously among patients. Some patients with MCI progress precipitately after the diagnosis, whereas a substantial proportion of patients does not develop dementia even after a prolonged period of up to 10 years [2]. In the stage of a clinically manifest dementia syndrome, studies have shown huge differences in rate of decline over time, even within a well-characterized cohort of persons with dementia of the Alzheimer type [3]. Understanding the causes leading to heterogeneous trajectories is essential for healthcare professionals to deliver personalized care and to maximally delay the progression of the condition. However, current knowledge cannot explain the phenotypical heterogeneity present in patients. Part of the heterogeneity in dementia presentation might be attributed to different etiologies, such as (the combination of) Alzheimer’s or vascular pathology. To further increase our understanding of the observed heterogeneity, a theoretical framework has been developed that posits dementia as a multi-causal, complex and dynamic disorder with involvement of many body systems/organs at different levels, rather than a disease with a single pathology [4]. The majority of patients with MCI and dementia are aged and may experience different levels of frailty and suffer from a considerable number of comorbidities that may affect clinical phenotype [5–9], How these patient characteristics influence the development and prognosis of the disorder is largely unknown. With the aim to evaluate the impact of comorbidity and frailty on the course of MCI and dementia, *the Clinical Course of Cognition and Comorbidity in Mild Cognitive Impairment and Dementia Study (4C study)* was conducted. The current paper outlines the study design, the study population and an overview of the data collected.

### 4C study research objectives

As part of the 4C study two complementary multicenter longitudinal cohorts were sampled in the Netherlands, with the following research objectives:

1. Explore heterogeneity in trajectories of progression in multiple outcomes, survival and institutionalization, as well as conversion rates (MCI to dementia and back and between different nosological subtypes) in persons with newly diagnosed MCI and dementia;
2. Investigate whether comorbidity (number and severity of chronic comorbidities and presence of certain comorbidities) and frailty influenced the course and outcome of MCI and dementia;
3. Investigate predictors of transitions (conversions, institutionalization, death) as well as compare outcome trajectories right before and after transitions.
4. Investigate predictors and outcome trajectories of persons who did not convert from MCI to dementia and who were not institutionalized
5. Develop prognostic rules for conversion and individual decline in cognition, behavior, and daily functioning, institutionalization and death.

These research objectives are studied both in the separate samples of MCI and dementia as well as in the combined sample to evaluate differences between these stages of increasingly severe cognitive decline.

## Construction and content

### Study design and participants

The MCI and Dementia cohort of the 4C study were similar in study design and implementation, but were conducted in different disease stages. Both cohorts aimed to enroll 300 participants at baseline, equally from the three participating centres Alzheimer Center of the VU University Medical Center Amsterdam, Alzheimer Centre Limburg of the Maastricht University Medical Center and Radboudumc Alzheimer Center, Radboud university medical center. The departments hosting the three memory clinics have a background in neurology (Amsterdam), old age psychiatry (Maastricht) and geriatric medicine (Nijmegen).

The study base of this cohort study were all persons referred to one of the three memory clinics by their primary care physician or referring medical specialist. The inclusion period ran from the March 2010 until May 2011. All consecutive referrals were prospectively evaluated for study eligibility during that period.

People with a new diagnosis of dementia made during this memory clinic visit were eligible for the Dementia cohort. Patients who had subjective cognitive decline or objective cognitive impairments based on neuropsychological test results, but did not fulfill the diagnostic criteria for dementia were eligible for the MCI cohort. The exact eligibility criteria for the 4C–MCI and the 4C–Dementia cohorts are shown in Table 1. As such the inclusion was broader than in most studies on MCI and dementia since comorbidities were allowed, thus resulting in a patient sample that was more representative for regular patient groups.

**Table 1:** Eligibility criteria for the 4C Study

Inclusion criteria	The 4C–MCI cohort	The 4C–Dementia cohort
• Cognitive performance	subjective cognitive complaints and/or objective cognitive impairments	objective cognitive impairments
• Fulfil the DSM-IV diagnostic criteria for dementia [12]	No	Yes
• Clinical Dementia Rating (CDR) Scale [34]	0–0.5	0.5–2
• Mini Metal State Examination (MMSE) score [35]	Not specified	≥10
• Age	≥ 55 years	Not specified
Exclusion criteria (similar for both cohorts)		
• The absence of a reliable informant (who has contact with the participant for at least once a week);		
• If the participant was expected unable to have at least one follow-up based on clinical judgment of the physician responsible for the care of the patient at the memory clinic;		
• The presence of other neurological disorders that could cause cognitive impairment or affect cognitive performance, such as Parkinson’s Disease, Huntington’s Disease, Normal Pressure Hydrocephalus (NPH), Korsakov’s syndrome, and a medical history of brain tumour, encephalitis or epilepsy. However, participants suffering from other cerebrovascular or psychiatric disorders were included in the studies.		

The clinician responsible for the care during the memory clinic visit asked whether patients would be willing to consider participation in the 4C study. If so, they were provided with written study information and a visit was scheduled with a research assistant to obtain written informed consent from the participant and an informant and to complete the baseline assessments. In order to be eligible, an informant had to be available and have contact with the participant for at least once a week. Almost all informants (about 90%) were either the participant’s partner or offspring, often also serving in a caregiving role. Not all participants had an informant who provided care that qualified as informal caregiving. However, if such a person could be identified, this person was asked as the informant. As a rule the same informant participated across the follow ups. In a small number of cases exceptions were allowed to minimize missings.

### Data collection

**Fig. 1:**
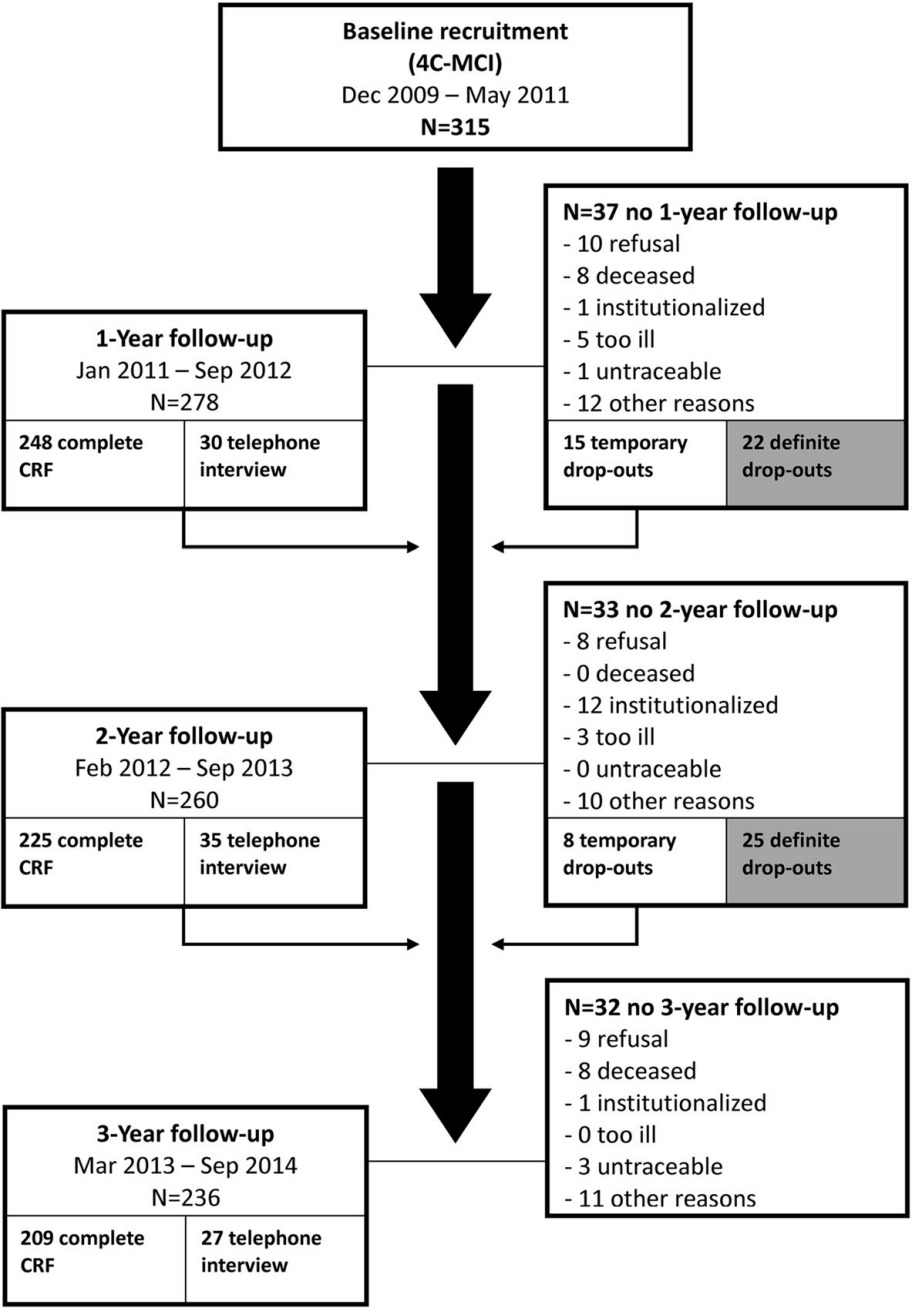
Flowchart of the 4C–MCI cohort

**Fig. 2:**
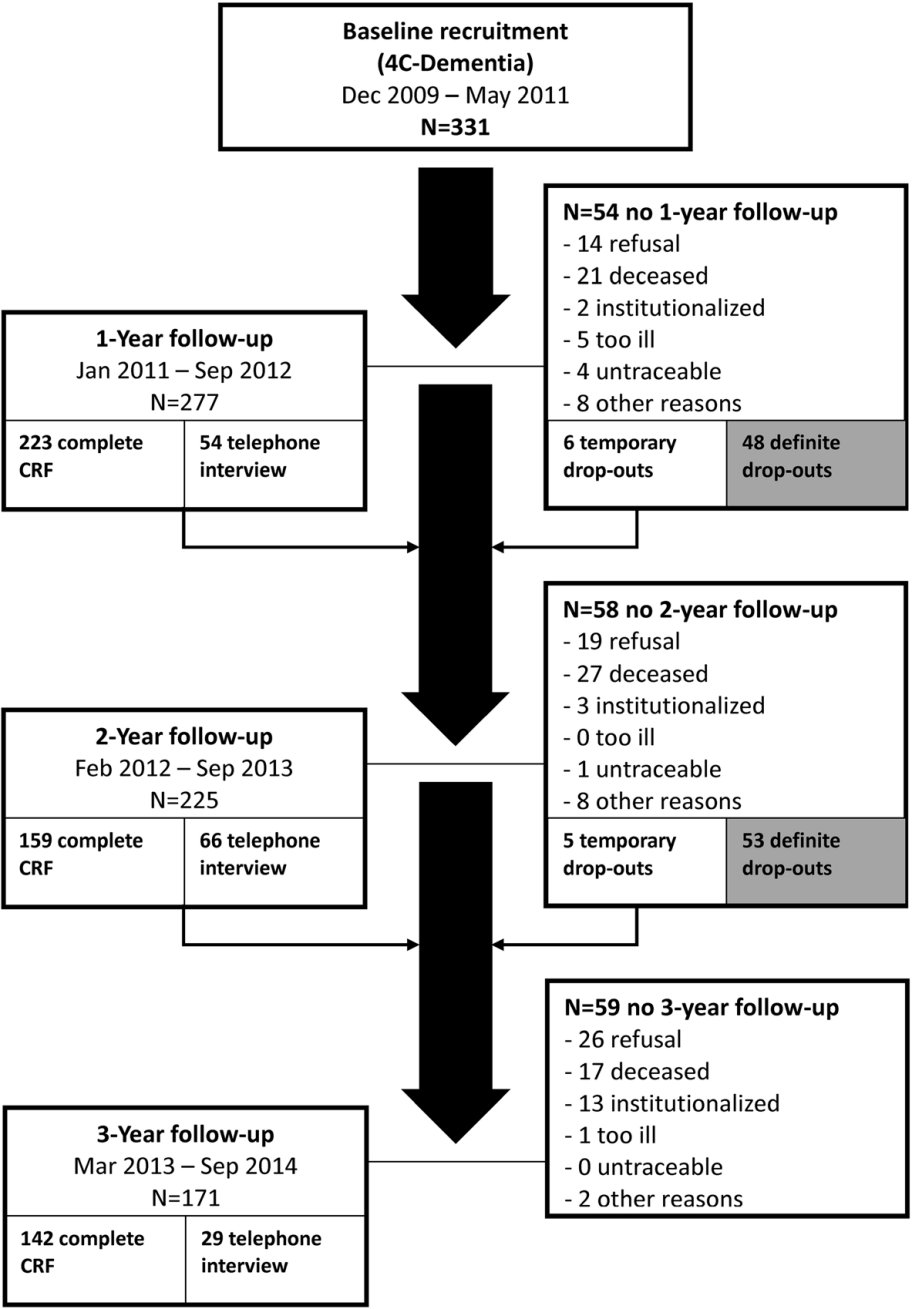
Flowchart of the 4C–Dementia cohort

The participants were scheduled to have three annual follow-ups after the baseline measurement during visits at the memory clinic. Annual follow-ups had to be conducted within a 3 months’ time frame around the dates of an annual schedule. The last assessment in the participants who last entered the study was conducted in August 2014. The flowcharts for both cohorts are shown in Figs. 1 and 2.

A multidisciplinary team consisting of physicians, neuropsychologists and research assistants collaborated in the standardized collection of the baseline and follow up data with the patient and the informant. As such, the multidisciplinary team (led by a consultant neurologist, psychiatrist or geriatrician respectively) responsible for the clinical care of the participant at the memory was also responsible for the research data collection. Data were collected on demographics, syndrome diagnosis, cognitive functioning, health conditions, functional abilities, neuropsychiatric symptoms, quality of life, and care resource use. An overview of the collected data per measurement, who was the source of the information and who collected the information is presented in Table 2. The assessor scored the reliability of the participant’s answers.

**Table 2:** Contents of data collection in the 4C studies

	Baseline	12 months	24 months	36 months	Assessed by	Source
Informed consent	√				Research assistant	Participant and informant
Demographics	√	√	√	√	Research assistant	Participant and informant
MMSE [35]	√	√	√	√	Physician	Participant
CDR [34]	√	√	√	√	Physician	Participant and informant
Subjective cognitive functioning [17]	√	√	√	√	Physician	Informant
Neuropsychological assessment	√	√	√	√	Psychologist	Participant
Physical examination & structural medical review	√	√	√	√	Physician	Participant and informant
Medications (prescribed & taken)	√	√	√	√	Physician	Participant and informant
Hospitalization (if any)		√	√	√	Research assistant	Participant and informant
Syndromal diagnoses	√	√	√	√	Physician	Participant and informant
Comorbidities (CIRS-G) [18]	√	√	√	√	Physician	Participant and informant
Frailty (Fried criteria + Frailty Index) [19, 20]	√	√	√	√	Research assistant	Participant and informant
DAD (disability assessment) [21]	√	√	√	√	Research assistant	Informant
GDS-15 (depression, self-reported) [22]	√	√	√	√	Research assistant	Participant
NPI (neuropsychiatric symptoms) [23]	√	√	√	√	Research assistant	Informant
Euroqol 5D [24]	√	√	√	√	Research assistant	Informant
Care resource use	√	√	√	√	Research assistant	Informant
Refusal or withdrawal data	√	√	√	√	Research assistant	Participant and informant
Institutionalization (outcome & date)		√	√	√	Research assistant	Participant and informant
Death (outcome & date)		√	√	√	Research assistant	Informant

In order to minimize the dropout rate, home visits by a researcher trained as a psychologist were implemented for those participants who were willing to continue participation in the study but unable to visit the memory clinics. In case participants needed an institutionalization, they continued with the study. The possibility to do the follow up assessment during a home visit also facilitated this.

#### Syndrome diagnosis of MCI and dementia

Syndrome diagnoses were made at baseline and reviewed at every follow-up visit based on clinical assessment by the physician and the multidisciplinary team. For the minority of cases in whom the follow up assessments were operationalized as a home visit, the diagnosis review was determined by a physician from the research data. The diagnosis of MCI was based on Petersen criteria [10] and was operationalized as a z-score lower than -1.5 SD (Dutch norm) on any of the cognitive tests (see below). Subjects with cognitive complaints without such a verified impairment on cognitive tests were categorized as subjective cognitive decline (SCD) [11]. The diagnosis of dementia was based on DSM-IV criteria [12]. Nosological dementia diagnoses were made according to standardized clinical criteria for AD (NINCDS-ADRDA criteria [13]), vascular dementia (NINDS-AIREN criteria [14]), frontotemporal dementia [15], and dementia with Lewy bodies [16].

#### Cognitive assessment

Data about the self-reported onset (gradual or sudden), course (stable, fluctuating or progressively deteriorated) and duration of the cognitive complaints were collected. In addition, patients were asked whether they had noticed a change in four cognitive functions (memory, concentration, mental capacity, and vitality) during the last year [17]. Change in each function was scored on a 7-point Likert scale ranging from -3 (very strong decline) to +3 (very strong improvement). If the participant was unable to answer by himself, the informant reported it based on daily contact and observation.

The neuropsychological assessment consisted of the same standardized battery of cognitive tests as used in the Dutch Parelsnoer Instituut (www.parelsnoer.org), covering the following domains: global cognition, episodic memory, implicit visual learning, working memory, word fluency, information processing speed, attention, executive functioning and visual perception (Table 3) [17].

**Table 3:** Standardized cognitive tests and the corresponding domains

Cognitive domain(s)	Neuropsychological tests
Global cognition	Mini Mental State Examination (MMSE) [35]
Episodic memory	15-Word Verbal Learning Test (VLT) immediate recall (5 trails), delayed recall and delayed recognition [36]
Implicit associative visual learning	Visual Association Test (VAT), short version [37]
Working memory	Digit Span subtest (forwards and backwards) of the Wechsler Adult Intelligence Test (3rd Edition) [38]
Verbal word fluency/semantic memory	60 s animal fluency [39]
Information processing speed	Letter Digit Substitution Test (LDST) [40]
Information processing speed, attention and executive functioning/response inhibition	Stroop Color Word Test (SCWT) [41]
Information processing speed, attention and executive functioning/concept shifting	Trail Making Test (TMT) parts A & B [42]
Visual perception	Visual Object and Space Perception battery (VOSP, Optional) Subtests of dot counting and incomplete letters [43]

#### Further assessments and questionnaires

Height, weight, and, waist circumference, systolic and diastolic blood pressure of the participants were recorded. Smoking and drinking status, the type, quantity and frequency of tobacco and alcohol consumption were asked. The physician structurally reviewed the patient’s personal medical history at baseline, and particularly queried extrapyramidal symptoms, gait disturbances, and family history. Newly diagnosed diseases were updated at every follow-up visit, also recording any taken medication and each hospitalization (Table 4).

**Table 4:** Structural medical review of participant’s health conditions

Body systems	Concerned diseases
Cardiovascular	angina, myocardial infarction, angioplasty/stent, coronary bypass surgery, carotid stenosis, hypertension, heart failure, others
Cerebrovascular	transient ischemic attack (TIA), cerebrovascular accidents (infarction, bleeding), reversible ischemic neurologic deficit (RIND), others
Endocrinal	diabetes mellitus, hypothyroidism, hyperthyroidism, obesity, others
Psychiatric	depression, psychosis, delirium, others
Somatic	chronic obstructive pulmonary disease (COPD), kidney problems, liver problems, others
Medications	the names, doses, frequencies and the indications of the medications
Hospitalization	the primary diagnosis, acute or routine medical care, the treating department, the total number of nights spent in the hospital

The Cumulative Illness Rating Scale for Geriatrics (CIRS-G) was used to further quantitatively measure disease burden and reflects the number and severity of the diseases [18]. Using CIRS-G, all diseases were classified into 14 organ systems: cardiac (heart only), vascular, hematologic, respiratory, ophthalmologic and otorhinolaryngologic, upper gastrointestinal, lower gastrointestinal, hepatic and pancreatic, renal, genitourinary, musculoskeletal and tegumental, neurologic, endocrine/metabolic and breast, and psychiatric. If a patient had multiple diseases in a single category, the most severe one was appraised. The severity of impairment was rated with a 0–4 grading scale from no problem to extremely severe problem (e.g. organ failure) resulting in a total score ranging from 0 to 56. The CIRS-G can be adapted as a measure of pure physical functioning in the 4C studies by excluding the psychiatric category, since this category includes cognition related comorbidities. The total score runs from 0 to 52.

Frailty was operationalized using the five physical criteria developed by Fried (2001) [19]. To that aim, grip strength (dominant hand, average of two measures) was measured using a Jamar® hand dynamometer and gait speed while walking at usual speed across 4.5 m (average of two measures). Unintentional weight loss and fatigue (two questions of Center for Epidemiologic Studies Depression Scale (CES-D)) were evaluated using questionnaires. Impaired physical activity was evaluated by asking whether the participant yes or no exercised, performed household chores, did odd jobs around the house or gardened. If they did any of these, this item was scored as not impaired. If participants did not engage in any of these activities they were asked to rate the time per week engaged in biking or walking. Multiplying these estimates by 4 and 3.5 kcal/min respectively and summing them resulted in the estimated weekly energy expenditure related to biking and walking. If this estimate was below 393 or 280 kcal/week for men and women respectively, physical activity was scored as impaired. A frailty index based on accumulated deficits also was operationalized from the 4C data [20].

The Disability Assessment for Dementia (DAD) scale, measuring both basic and instrumental activities of daily living (ADL, IADL), was used to evaluate the severity of impairment in everyday functioning through a structured interview with the informant [21]. The participants’ abilities to perform ten different (I)ADL activities in the 2 weeks prior to the assessment were rated. The final DAD score was converted to a percentage from 0 to 100, in which a higher percentage indicates a higher level of daily functioning.

The 4C study used the Geriatric Depression Scale (GDS-15) to assess the presence and severity of depressive symptoms in older persons [22]. The Neuropsychiatric Inventory (NPI) was used to characterize the psychopathology of the patient through a structured interview with the informant, assessing 12 common neuropsychiatric symptoms [23]. A screening question for each neuropsychiatric symptom was asked to confirm the presence of the symptom before rating the frequency (1–4, rarely–very often), severity (1–3, mild–severe), and burden (0–5, not at all-extremely) for the informant. The total NPI score ranged from 1 to 144.

The EQ-5D was used to measure health-related quality of life and results in a score of 0–100, where higher scores indicate better functioning [24]. The EQ-5D was rated by the patient and by the informant: for the situation of the patient and for the informant him-/herself.

Besides patient and disease related data, also data about the patient’s care resource use was investigated by assessing the number of visits to the general practitioner, home care, day care, institutionalized care, hospital in- and outpatient, emergency care, other professionals, informal care, medical goods, and out-of-pocket expenditures using an informant questionnaire.

#### Refusal or withdrawal, minimum but essential data collected for research

The reasons for dropout were collected and categorized. The attrition is shown in the flowchart of Figs. 1 and 2. With their consent, the study investigators made efforts to collect minimal but essential study data through a telephone interview when the participant (or informant) wanted to discontinue their full participation. Researchers asked about the course of cognitive symptoms, functional abilities in (I)ADLs (any help or assistance needed in certain activities), and global severity of dementia (CDR). If the participant had received a diagnosis of MCI or dementia from another physician, the syndrome diagnosis was documented. Despite the participant’s refusal to participate in a particular follow-up assessment, some patients agreed to be approached at the next follow up and did return to fully participate in the studies, as shown in Figs. 1 and 2.

#### Adaptations of study protocol and implementation

Some changes to the original research protocol were made for better implementation and completion of the studies. Based on the experiences of baseline interview, an evaluation of the reliability of information provided by the participant and the informant was added to the follow up assessments.

As a default, the assessments were carried out in the memory clinics. However, for some participants, it was inconvenient to visit the memory clinic due to immobility or other physical conditions. Where possible, home visits (to the patient’s house or nursing home) were implemented in the follow-ups and carried out by a well-trained researcher, to limit study attrition.

## Utility and discussion

### Results

In the MCI and dementia cohort, 315 and 331 participants were included respectively. Noticeably, more men than women were recruited in the MCI cohort (Table 5), consistently across the three centers, whereas more women were recruited in the Dementia cohort, except in Amsterdam. Participants in the MCI cohort were 5.2 years younger, and had better cognitive performance, functional abilities, physical condition and quality of life, but had higher depression scores on average than those in the Dementia cohort at baseline (Table 6).

**Table 5:** Baseline demographic characteristics of the participants in the 4C Study

*N* (%), unless stated differently	4C-MCI cohort (*n* = 315)	4C-Dementia cohort (*n* = 331)
Age at baseline (years, mean ± SD)	69.7 ± 8.5	74.9 ± 10.2
Female sex	111 (35)	182 (55)
Caucasian	311 (99)	326 (98)
Education		
Low (lower than middle school)	126 (40.0)	142 (44.1)
Middle (high school/vocational education)	78 (24.8)	90 (28.0)
High (university)	111 (35.2)	90 (28.0)
Marital Status		
Married/registered partnership, cohabiting	244 (77.5)	202 (61.2)
Widow/widower	42 (13.3)	93 (28.2)
Single, divorced	29 (9.2)	35 (10.6)
Living situation		
Alone, independently	59 (18.7)	100 (30.5)
With partner and/or children	242 (76.8)	205 (62.5)
Institutionalized living	9 (2.9)	21 (6.4)
Others	5 (1.6)	2 (0.6)
Informant’s relation with the participant		
Partner	224 (71.1)	179 (54.4)
Offspring	60 (19.5)	115 (35.0)
Sibling	7 (2.2)	10 (3.0)
Other relative or acquaintance	23 (7.3)	25 (7.6)

**Table 6:** Baseline cognitive, physical and functional characteristics and quality of life of the participants in the 4C Study

Mean ± SD, unless stated differently	4C-MCI cohort (*n* = 315)	4C-Dementia cohort (*n* = 331)
Subclassification cognitive disorder, *N* (%)^a^		
Subjective cognitive decline	90 (29)	n.a.
Amnestic MCI	151 (48)	n.a.
Non-amnestic MCI	71 (23)	n.a.
Alzheimer’s disease (probable or possible)	n.a.	216 (65)
Vascular dementia or any dementia diagnosis with a vascular component	n.a.	71 (21)
Any other dementia diagnosis without a vascular component	n.a.	44 (13)
Self-reported duration of cognitive problems (years)	3.3 ± 3.8	3.1 ± 2.6
Self-reported course of cognitive problems, *N* (%)		
Progressive	213 (67.6)	285 (89.1)
Stable	40 (12.7)	14 (4.4)
Fluctuated	33 (10.5)	21 (6.6)
Cognitive functions^b^		
MMSE (range 0–30, lower is worse)	26.9 ± 2.6	21.9 ± 3.7
VLT immediate recall (z-score, lower is worse)	−1.1 ± 1.2	−2.3 ± 1.1
VLT delayed recall (z-score, lower is worse)	−1.2 ± 1.3	−2.4 ± 0.9
60 s animal fluency (z-score, lower is worse)	−0.7 ± 0.8	−1.7 ± 0.9
TMT-A (z-score, lower is worse)	−0.3 ± 1.4	−1.7 ± 1.9
TMT-B (z-score, lower is worse)	−0.4 ± 1.3	−1.8 ± 1.4
SCWT card 1 + 2 (z-score, lower is worse)	−1.2 ± 1.7	−2.8 ± 2.5
SCWT card 3 (z-score, lower is worse)	−1.2 ± 2.4	−3.5 ± 3.2
Neuropsychiatric symptoms		
GDS-15 (range 0–15, higher is worse)	3.6 ± 2.8	3.1 ± 2.7
NPI (range 0–144, higher is worse)	14.5 ± 15.0	16.3 ± 16.3
BMI (kg/m^2^, mean ± SD)	26.2 ± 3.9	25.9 ± 4.3
DAD (range 0–100, lower is worse)	86.7 ± 15.7	70.8 ± 24.1
CIRS-G		
Total score (range 0–52^c^, higher is worse)	7.09 ± 4.87	7.48 ± 4.94
Severity index (total score/number of categories endorsed, range 0–4, higher is worse)	1.57 ± 0.51	1.63 ± 0.55
Comorbidity index (number of categories with score ≥2, range 0–13, higher is worse)	2.25 ± 1.93	2.37 ± 1.90
CIRS-G category with score ≥2 (at least moderate disability or morbidity/requires “first line” therapy), *N* (%)		
Heart	92 (29.2)	90 (27.3)
Vascular	144 (45.7)	170 (51.5)
Hematopoietic	12 (3.8)	25 (7.6)
Respiratory	74 (23.5)	54 (16.4)
Eyes/ears/nose/throat	54 (17.1)	72 (21.8)
Upper gastrointestinal	48 (15.2)	43 (13.0)
Lower gastrointestinal	28 (8.9)	35 (10.6)
Liver	20 (6.3)	21 (6.4)
Renal	15 (4.8)	19 (5.8)
Genitourinary	57 (18.1)	75 (22.7)
Neuromuscular	40 (12.7)	70 (21.2)
Neurological	75 (23.8)	48 (14.6)
Endocrine	49 (15.6)	59 (17.9)
Frailty according Fried Criteria, *N* (%)		
Not frail (≤1 of 5 criteria satisfied),	219 (74.7)	224 (68.9)
Pre-frail (2 of 5 criteria satisfied)	44 (15.0)	55 (16.9)
Frail (≥3 of 5 criteria satisfied)	30 (10.2)	46 (14.2)
EQ-5D (with Dutch weights) (range 0–1, lower is worse)	0.98 ± 0.06	0.81 ± 0.20
EQ-5D VAS (range 0–100, lower is worse)	69.4 ± 15.6	67.5 ± 16.9

^a^3 (1% of 315) participants in the 4C MCI cohort with MCI could not be classified as amnestic versus non-amnestic MCI due to missing 15-Word Verbal Learning Test scores

^b^Despite the application of the Petersen criteria for definition of MCI, mean cognitive scores on the different domains may be above -1.5 SD of the Dutch norm scores, because people with SCD were included in the 4C MCI cohort, but do not satisfy the MCI criteria, also MCI patients were not required to score below -1.5 SD on all domains assessed

^c^the range of the CIRS-G is 0–52 rather than 0–56, as the psychiatric domain was left out of the scale to create a measure of purely physical comorbidity

Of 315 participants in the 4C-MCI cohort, 90 (29%) had subjective cognitive decline and 151 (48%) had amnestic MCI. Of 331 participants in the 4C dementia cohort, 216 (65%) had probable or possible AD. The majority of the participants experienced a progressive cognitive decline in the phase before the baseline diagnosis, namely 68% in the MCI cohort, and 89% in the Dementia cohort, starting at about 3–3.5 years on average before entering in either cohort. Episodic memory was the most severely impaired cognitive domain among MCI patients, whereas among dementia patients, multiple cognitive functions were severely impaired, especially executive functioning, attention and information processing speed.

By quantitative measures of disease burden (CIRS-G), vascular diseases were the most frequent and most severe comorbid category in both cohorts. The total CIRS-G score leaving out the category of psychiatric illness was higher in the dementia than in the MCI cohort. For the AD patients in the dementia cohort, it was found that comorbidities were associated with cognitive performance; frailty was associated with functional abilities at baseline [25]. Comparing older and younger persons with dementia contrasting persons from the 4c Dementia cohort data with persons from the NeedYD cohort [26], showed that younger persons had less comorbidity and may more often have neurological comorbidities than older persons with dementia [27]. Further studies into the relationship of cognitive decline with comorbidity and frailty are underway. In addition to the objectives described earlier, these will focus on determinants of health-related quality of life and gait speed and grip strength as predictors of cognitive decline in the MCI cohort.

The most prevalent neuropsychiatric symptoms reported by the informants in the MCI cohort were irritability (51%, average score 5.4 out of 12 in NPI), apathy/indifference (44%, 5.3) and depression/dysphoria (41%, 4.7). These neuropsychiatric symptoms were also most common in the dementia cohort, with different rankings: apathy/indifference (51%, 6.5), depression/dysphoria (38%, 5.3), irritability (36%, 5.3). When it occurred, agitation and aggression (mean 3.3; SD 1.13) was most burdening for informants in the MCI cohort. This was also the most burdening symptom in the Dementia cohort (3.0 ± 1.3).

After 3 follow-ups, 23 subjects (23/90, 26%) classified as SCD at baseline were reclassified as MCI, 23 subjects (23/225, 10%) with MCI reverted to SCD, and 74 subjects (10 subjects with SCD at baseline and 64 subjects with MCI at baseline) progressed to different types of dementia (Table 7). The annual incidence of MCI subjects progressing to dementia was 11% (32/278) in the first 12 months, 7% (18/260) between 12 and 24 months and 10% (24/236) between 24 and 36 months of follow up. In the dementia cohort, 91 patients (27%) received at least one different (nosological) diagnosis of dementia during the follow-ups. Of these 91, three participants were rediagnosed as MCI, and one participant went from dementia to MCI (12 m) and via again dementia (24 m) to SCD (36 m). Thus, in 4 (1.2%) of 331 participants the dementia diagnosis was reconsidered. 16 patients (5%) in the MCI cohort and 75 (23%) in the Dementia cohort died during 36 months of follow-up. Studies to develop and validate prognostic rules from these data are underway.

**Table 7:** The incidence of MCI subjects progressing to dementia within the study duration

Study time line	Number of subjects		%
	MCI conversion to dementia	In the cohort	
1 year follow-up	32	278	11.5
2 year follow-up	18	260	6.9
3 year follow-up	24	236	10.1
Total	74	315	23.4

### What are the main strengths and weakness?

One of the main questions for the 4C study was whether the interaction with health status would influence the natural disease course of cognitive disorders, and contribute to the heterogeneity of clinical outcomes. Thus, including participants with comorbidity in the study and getting reliable diagnoses of comorbidities were essential strengths of our studies, leading to more representative patient groups. Prospectively sampling diverse participants with natural disease courses from various routine clinical settings (neurology, geriatrics and old age psychiatry departments) who were representative for the MCI and dementia patients regularly seen in memory clinics was another strength. Finally, cognitive functioning is a central feature of MCI and dementia. In the 4C study, in addition to global measures of cognition, a series of standardized and validated neuropsychological tests were available to detail how different cognitive domains changed and to explore the effect of comorbidities on cognitive functioning in different domains over time. While some longitudinal studies focusing on risk factor discovery for dementia onset continue to follow persons who develop MCI and/or dementia, dedicated follow up studies are indispensable to understand the more advanced stages of cognitive decline. As such, the 4C study complements other population-based and clinical MCI and dementia follow up cohorts. In general, population-based (e.g. [28] or [6]) and clinical samples of MCI and dementia (e.g. [29] or [30]) are complementary in that the population-based studies have the advantage of being systematically sampled from well characterized base populations, whereas the clinical samples are able to add more detail in terms of frequency and type/comprehensiveness of assessment. To maximize the combined value of these data [31], initiatives such as IALSA [32] and COSMIC [33] help to identify datasets as well as provide methodologies in their combined analyses.

Discerning typical disease trajectories based on long-term observation and developing prediction models for disease progression would be beneficial for patient care. However, for frontotemporal dementia, dementia with lewy bodies or other types of dementia, due to limited cases, more effort should be made to include more patients in future studies to increase the power or combining the current samples with other studies.

Attrition was the biggest challenge for the 4C study, although it is inevitable due to the nature of the disease and the aged population (see Figs. 1 and 2). In the MCI cohort, 225 of 315 (71%) participants completed all three follow ups (209; 66%) or completed follow ups until the endpoint of death (16; 5%). In the Dementia cohort, 207 of 331 (63%) participants completed all three follow ups (142; 43%) or completed follow ups until the endpoint of death (65; 20%). Another 10 participants died during the 36 months of follow up, but dropped out of the study for one of the other reasons.

## Conclusions

Sampled in a clinical setting, the 4C study database complements population-based studies on neurodegenerative disorders in terms of the type of assessment (e.g. comorbidity, frailty, and functional status were repeatedly assessed). The 4C study complements available databases of clinical cohorts of MCI and dementia patients, because the exclusion criteria were kept to a minimum, to obtain a sample that is representative for the average patient visiting a memory clinic. Cognition, functional abilities, resource use and quality of life were the key outcome domains and their longitudinal changes and dynamic relations will be further explored, also related to endpoints such as death and institutionalization.

## Abbreviations

(I)ADLs: (Instrumental) activities of daily living
4C Study: The Clinical Course of Cognition and Comorbidity in Mild Cognitive Impairment and Dementia Study
AD: Alzheimer’s disease
CDR: Clinical Dementia Rating scale
CIRS-G: Cumulative Illness Rating Scale for Geriatrics
DAD: Disability Assessment for Dementia
EQ5D: EuroQoL-5D
GDS-15: Geriatric Depression Scale-15
MCI: Mild cognitive impairment
MMSE: MiniMental State Examination
N.A.: Not applicable
NPI: Neuropsychiatric Inventory
SCD: Subjective cognitive decline
SCWT: Stroop Color Word Test (3 subtasks)
SD: Standard deviation
TMT: Trail Making Test (part A & B)
VAS: Visual Analogue Scale
VLT: 15-Word Verbal Learning Test
